# Barriers and Enablers Influencing the Implementation of Artificial Intelligence for Diabetic Retinopathy Screening in Clinical Practice: A Scoping Review

**DOI:** 10.1111/ceo.14567

**Published:** 2025-06-14

**Authors:** Jenny Tran, Jose J. Estevez, Natasha J. Howard, Saravana Kumar

**Affiliations:** ^1^ College of Medicine and Public Health Flinders University Adelaide Australia; ^2^ Department of Ophthalmology, Flinders Centre for Ophthalmology, Eye and Vision Research Flinders University Adelaide Australia; ^3^ Caring Futures Institute, College of Nursing and Health Sciences, Optometry and Vision Science Flinders University Adelaide Australia; ^4^ South Australian Health and Medical Research Institute Adelaide Australia; ^5^ Faculty of Health and Medical Sciences University of Adelaide Adelaide Australia; ^6^ UniSA Allied Health and Human Performance University of South Australia Adelaide Australia

**Keywords:** artificial intelligence, barriers, diabetic retinopathy, enablers, implementation science

## Abstract

**Background:**

Diabetic retinopathy is a leading cause of preventable blindness worldwide. Meanwhile, artificial intelligence is rapidly growing in clinical utility within medicine. This scoping review aims to identify and summarise existing literature on the barriers and enablers of clinical applications of artificial intelligence systems for the screening of diabetic retinopathy.

**Methods:**

Utilising a systematic approach and the PRISMA‐ScR protocol for conducting scoping reviews, searches were performed in MEDLINE, Embase, Emcare, Cochrane, CINAHL, ProQuest, Scopus and grey literature (Australian Indigenous Health InfoNet). Two reviewers independently reviewed the records. A third reviewer provided consensus. Data extraction and synthesis in narrative form ensued.

**Results:**

A total of 3844 articles were screened, of which 18 were selected. Published between 2018 and 2023, the selected studies varied in study design and were conducted across 10 countries. Several barriers and enablers were identified and categorised into four domains: healthcare system, healthcare professional, healthcare user and information technology. Within the healthcare system, clinical efficiency was reported on most frequently. Concerning the healthcare professional, education was most frequently discussed. Within healthcare user, studies most frequently identified factors pertaining to patient outcomes, while diagnostic performance was most frequently explored under the information technology domain.

**Conclusions:**

As evidence for the efficacy of artificial intelligence for diabetic retinopathy screening grows, barriers to and enablers for its uptake in clinical practice are paramount considerations. Translating the knowledge of systems, provider, consumer and technological factors informs clinical strategies, ultimately facilitating the sustainable and effective implementation of this novel technology for screening practices.

## 1 Introduction

The International Diabetes Federation reported that approximately 537 million adults were living with diabetes in 2021, with three in every four cases living in low‐ and middle‐income countries [1]. The number of people with diabetes is projected to rise to 783 million by 2045 [1]. A microvascular complication of diabetes, diabetic retinopathy (DR) is globally a leading cause of visual loss and blindness [2]. The global reported prevalence of DR among people with diabetes is 23%, varying geographically from 17% in South‐East Asia to 36% in Africa [3]. As of 2020, this equates to an estimated 103 million adults, which is projected to rise to 161 million individuals by 2045 [3]. Higher prevalence of disease in lower‐resource settings and a scarcity of eye specialists proportional to screening needs are among the factors contributing to the DR burden [4, 5]. Despite the rise in diabetes and DR prevalence, the early detection and diagnosis of DR alongside the timely implementation of established treatment options may alleviate the associated morbidity.

The diagnosis of DR is possible through clinical assessment of the retina. Artificial intelligence (AI) systems are an emerging tool in ophthalmology, enabling the analysis of such retinal images to screen for DR and stage its severity. Several algorithms exist and are known to report clinically acceptable diagnostic accuracy [6, 7, 8]. The advancements in AI technologies confer great potential to revolutionise ophthalmic care, especially in contexts hindered by barriers such as geographically limited access to healthcare and resource constraints. Therefore, an opportunity exists to deploy eye health screening as a public health initiative through promoting the uptake of automated screening on a large scale, especially in hard‐to‐reach populations and community‐based primary provider settings. The reformation of eye health services acts to moderate the social determinants of health, thereby offering an avenue to address eye health inequities for underserved and priority populations [9].

While research has been published to date on the diagnostic capabilities of AI models for the detection of DR, factors influencing the application of AI for DR screening in clinical settings are not widely reported [6, 7, 8, 10]. Therefore, the purpose of this review is to address this knowledge gap by identifying the barriers and enablers influencing the applicability of AI systems for DR screening in clinical settings. The findings from this research can facilitate the implementation of novel AI technologies for DR screening.

## 2 Methods

A scoping review methodology was selected to establish the scope of the literature in the domain of AI used for DR screening in healthcare settings. The PRISMA‐ScR guidelines directed the protocol for this study [11]. A pre‐specified scoping review protocol was established and is available on the Open Sciences Framework (https://osf.io/t2xc4/) [12]. The conduct and reporting followed the framework proposed by Arksey and O'Malley [13].

### 2.1 Identifying the Research Question

The scoping review sought to gain insights into the following research question: ‘*What are the barriers and enablers influencing the applicability of AI for DR screening in clinical practice?*’

### 2.2 Inclusion Criteria

Studies reporting the application of AI technologies utilised for DR screening in a clinical setting were eligible for inclusion. This included both published and unpublished studies.

### 2.3 Exclusion Criteria

Opinion articles and discussion papers were excluded. Studies reporting the use of AI in medicine for non‐DR applications were ineligible. Studies reporting only the efficacy of AI for DR screening, for example, sensitivity, specificity and receiver operating curves (AUC), were not included in the review. Studies were excluded if not authored in the English language.

### 2.4 Identifying Relevant Studies

A search strategy was developed for MEDLINE (Appendix A) under the guidance of a research librarian. This strategy was modified for additional searches conducted in the following databases: Embase, Emcare, Cochrane Library, CINAHL, ProQuest and Scopus. A further search was conducted on Australian Indigenous Health InfoNet to identify sources of grey literature and sources pertaining to Aboriginal and Torres Strait Islander health in Australia. Limitations were not applied to the searches. The results were exported and saved to EndNote, where sources were deduplicated and subsequently imported to Covidence for a second screen of duplicates. Eligibility criteria informed the selection of studies by reviewers.

### 2.5 Screening

Researchers collaboratively reviewed articles using the review software Covidence. J.T. and J.J.E. independently screened all retrieved sources by title and abstract, using the inclusion and exclusion criteria. N.J.H. resolved the conflicts from this process to determine articles eligible for full‐text review. Researchers J.T. and J.J.E. independently conducted full‐text reviews of the remaining articles, and N.J.H. resolved conflicts to determine the sources eligible for final inclusion.

### 2.6 Data Extraction

J.T. extracted data from selected articles using a data extraction table, informed by the research expertise within the team and a literature review on barriers and enablers in implementation science. Items included in the extraction sheet were author(s), year, country, study design, population, intervention, setting, barriers and enablers reported and limitations of the study. K.Z‐A. verified the data extraction for 20% (*n* = 4) of those selected. Per scoping review protocols, the selected articles were not appraised for quality or methodology.

### 2.7 Collating, Summarising and Reporting

Data extracted from each paper were collated and themes were identified within the results. Barriers to the application of AI for DR screening were elucidated alongside enablers, under the domains of healthcare system, healthcare professional, healthcare user and information technology as informed by the experience of researchers on the team. Duplicate or irrelevant items were discarded following discussion among researchers. Overall, the data were synthesised in narrative form.

## 3 Results

### 3.1 Search Results

**FIGURE 1:**
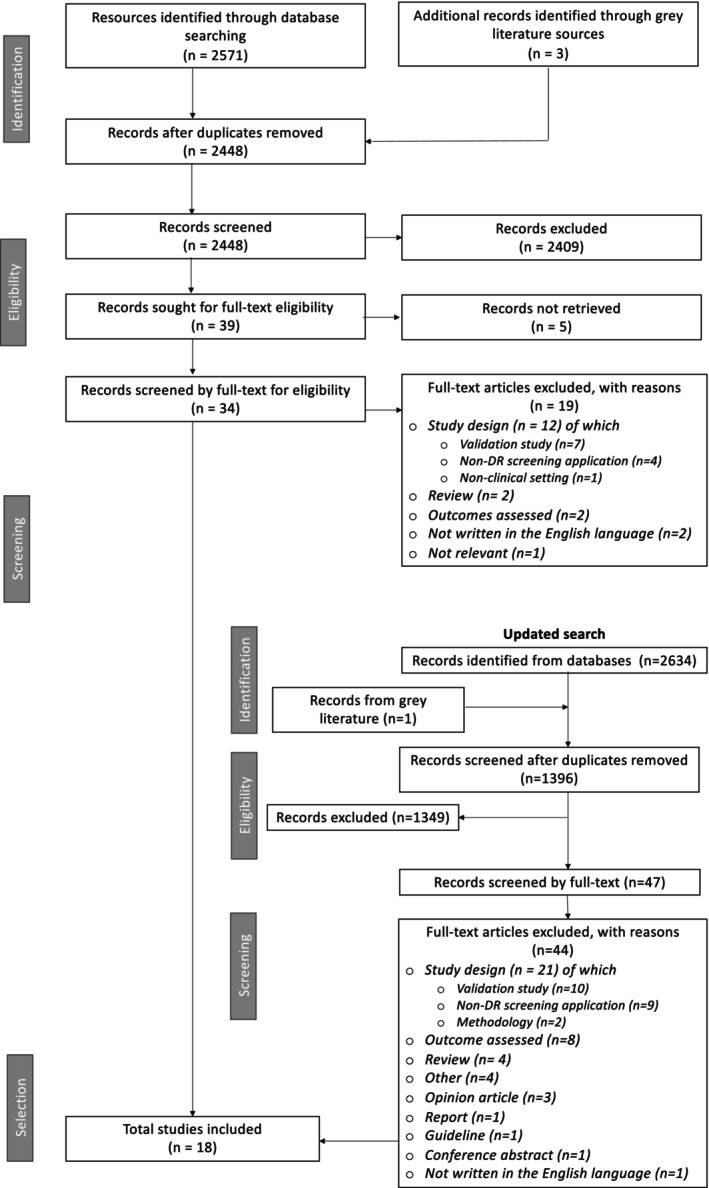
Flow chart of study selection process.

The initial search was conducted on 2 March 2022 and identified 2574 records (Figure 1). After the removal of duplicates, a total of 2448 articles were screened by title and abstract, of which 2409 articles did not meet the inclusion criteria; the 39 remaining papers were screened in full text. Out of these articles, five records were not retrieved due to being conference abstracts, 19 were excluded, and 15 were included. An updated search was conducted on 21 January 2023 yielding 1396 records after de‐deduplication. Screening by title and abstract excluded 1349 articles, and the remaining 47 papers were screened in full text, of which 44 were excluded. From this search, three articles were included. Overall, 18 articles met the criteria for inclusion.

### 3.2 Description of the Included Studies

The 18 included studies were published between the years 2018 and 2023 (Table 1). Most studies were conducted in Australia (*n* = 4; 22%). Second most frequent were studies conducted in the USA (*n* = 3; 17%), followed by China (*n* = 2; 11%), Germany (*n* = 2; 11%) and New Zealand (*n* = 2; 11%), then Saudi Arabia, Sri Lanka, Taiwan, Thailand and Zambia (each *n* = 1; 6%) [14, 15, 16, 17, 18, 19, 20, 21, 22, 23, 24, 25, 26, 27, 28, 29, 30, 31]. All studies were contextualised in a clinical setting, and three studies (17%) did not deploy an AI system for DR screening. Many study designs comprise the articles selected, including prospective cohort (*n* = 7) [17, 19, 20, 23, 24, 26, 31], survey (*n* = 3) [14, 16, 28], clinical validation (*n* = 3) [15, 22, 30], multi‐method (*n* = 2) [25, 29], cross‐sectional (*n* = 1) [21], retrospective (*n* = 1) [27] and diagnostic (*n* = 1) [18].

**TABLE 1:** Characteristics of included studies.

Author (References)	Paper type	Country	AI intervention	Clinical setting
Barakat [14]	Cross‐sectional study using self‐administered online‐based questionnaire	Saudi Arabia	—	Workplaces employing ophthalmologists, family physicians, endocrinologists, and general physicians involved in diabetic eye care
Bellemo et al. [15]	Clinical validation study	Zambia	Ensemble AI technology: VGGNet + ResNet	Mobile screening units of five urban centres
Held et al. [16]	Qualitative study	Germany	—	General practitioners, medical assistants in primary care, and an ophthalmologist
Ipp et al. [17]	Prospective multi‐centre cross‐sectional diagnostic study	USA	EyeArtAutomated DR Detection System, version 2.1.0 (AI)	15 clinics across primary care, general ophthalmology and retinal specialty centres
Kanagasingam et al. [18]	Diagnostic study	Australia	AI system using deep‐learning Inception v3 as the base model	Metropolitan primary care practice
Keel et al. [19]	Prospective pilot study	Australia	Two deep learning models using inception‐v3 architecture	Two urban outpatient endocrinology departments
Li et al. [20]	Cross‐sectional study	Taiwan	A Deep Learning algorithm using Inception V4 as the base model	Endocrinology outpatient department at a hospital
Liu et al. [21]	Prospective cohort study	USA	EyeArt 2.0	Primary care clinic
Mehra et al. [22]	Validity and reliability analysis	USA	IDx‐DR AI	Primary care clinic
Ming et al. [23]	Prospective, cross‐sectional real‐world diagnosis validation study	China	EyeWisdom	Primary care clinic
Ruamviboonsuk et al. [24]	Prospective interventional cohort study	Thailand	A deep‐learning AI system	Nine primary care sites under Thailand's national diabetic retinopathy screening programme (eight rural community hospitals + one urban primary care clinic in tertiary care hospital)
Scheetz et al. [25]	(1) Prospective observational study + (2) survey	Australia	Inception‐v3	Two endocrinology outpatient settings + three Aboriginal Medical Services clinics
Vaghefi et al. [26]	Retrospective study	New Zealand	THEIAsystem	Datasets from two large district health boards
Vaghefi et al. [27]	Prospective multi‐centre trial	New Zealand	THEIA	Participants from (1) one urban eye hospital + (2) one semi‐rural optometrist‐led screening provider
Watson et al. [28]	Qualitative study	Australia	—	15 general practitioners recruited from urban and rural general practices
Wijesinghe et al. [29]	(1) Retrospective validation study + (2) qualitative study using a scale to assess usability	Sri Lanka	An Intelligent Diabetic Assistant comprising 4 submodules	Retinal dataset collected from an eye hospital in Sri Lanka + recent Kaggle competition
Wintergerst et al. [30]	Clinical validation	Germany	EyeArt, version 2.1.0	General practice
Zhang et al. [31]	Multicentre prospective study	China	VoxelCloud Retina	155 diabetes centres in China

### 3.3 Findings

**FIGURE 2:**
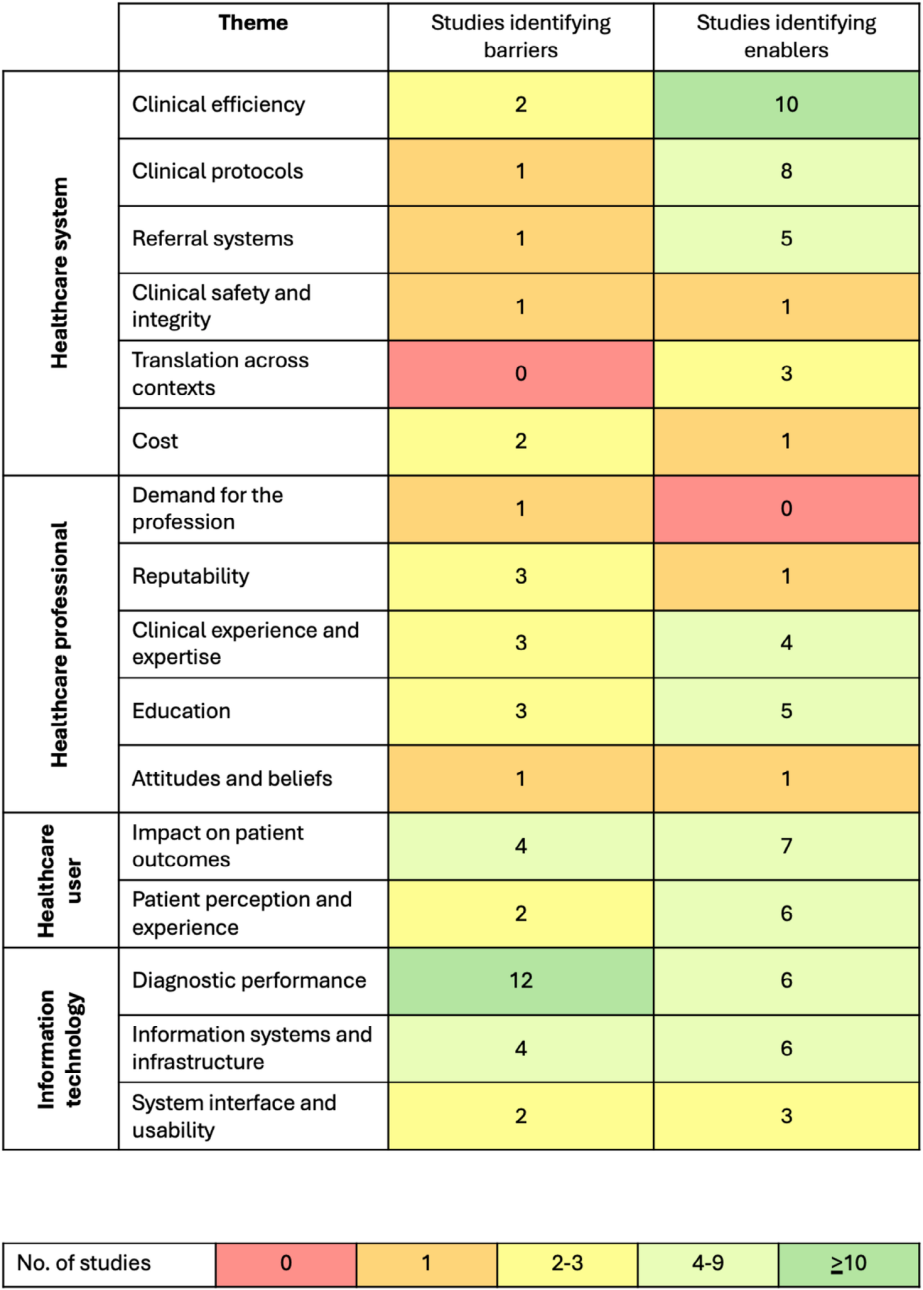
Frequency of barriers and enablers discussed by studies categorised by theme and domain.

Barriers and enablers were classified into four domains (Figure 2). The *healthcare system* domain referred to the health system broadly and its various components. The *healthcare professional* domain referred to the individual clinician and the *healthcare user* domain referred to patients and consumers of healthcare and their perspectives. The *information technology* domain referred to the technology itself and its use in healthcare contexts.

### 3.4 Healthcare System

#### 3.4.1 Clinical Efficiency

Clinical efficiency was described in several studies. The time to return a diagnosis after retinal imaging was deemed a barrier when it took 3 days in one study, and an enabler when immediate in seven studies [15, 21, 22, 23, 25, 26, 27, 30]. One study described that point‐of‐care image analysis and results generation enabled suspected pathology to be further imaged with a different modality (i.e., optical coherence tomography) during the consultation, followed by immediate telehealth recruitment as appropriate, preventing the need for the patient to return for an additional appointment [27]. Three studies reported that integrating the AI system was time‐consuming [24, 25, 30]. Conversely, it was subjectively reported by Barakat that 79% (*n* = 244/309) of participants believed AI would increase clinical efficiency, with the reduction in clinician workload an enabler in Wijesinghe et al. [14, 29]. One study described the potential for AI technology to enable earlier escalation to a doctor for detected abnormalities [16]. Regarding imaging, time to image capture was reported in three studies; Kanagasingam et al. cite between 10 and 15 min to obtain a colour fundus photograph, Keel et al. report a mean time of 7 min to conduct AI screening, and Wintergerst et al. report the time to capture images of both eyes was less than 10 min for 85% (*n* = 64/75) of patients [18, 19, 30]. The number of attempts was variable, ranging from one to five in one study [30]. Finally, one study reported that a paper‐based system inhibited the ability to track patient referrals [24].

#### 3.4.2 Clinical Protocols

The administration of mydriatics was expressed as a potential barrier in one study reporting that of all AI‐gradable fundus photos, 55% (*n* = 580/1052) of images were gradable without pupillary dilation [22]. Meanwhile, four studies reported the use of non‐mydriatic fundus photographs as an enabler, minimising the need for administration of medical mydriatics to adequately visualise the retina [17, 24, 27, 31]. Specifically, the physical clinic setting was a barrier in four papers [18, 20, 21, 30]. Uneven light exposure and peripheral light artefacts were barriers to practical implementation in three studies [18, 21, 30]. In one study, despite standardisation processes wherein all photos were taken by a trained technician in a dark room, 69% (*n* = 981/1432) of fundus photos were of sufficient quality for analysis [20]. Control of environmental factors was reported in one study, which described the use of a light‐tight fabric as a strategy to reduce artefactual ambient light [30].

#### 3.4.3 Referral Systems

One study reported, as a barrier, an over‐estimate of ungradable images by the AI technology compared to retinal specialists; retina specialist over‐readers and an adjudication panel of retinal fellows believed only 64% (*n* = 387/602) and 58% (*n* = 236/409), respectively, of images that were referred were indeed referrable [24]. Five studies described efficient referrals as favourable [17, 18, 21, 24, 25]. More efficient use of eye‐care services and a reduced burden on practitioners were reported in two studies [21, 25].

#### 3.4.4 Clinical Safety and Integrity

Safe access to healthcare was explored in one qualitative study, reporting that 86% (*n* = 264/309) of respondents agreed with a statement that AI will minimise non‐essential contact between eye‐care providers and health users in a post‐pandemic context [14]. One study identified data security concerns from health professionals relating to smartphone use [16]. Meanwhile, 75% (*n* = 232/309) of respondents in one study agreed that AI can maintain patient confidentiality [14]. One study reported suspicion held by healthcare workers relating to the commercial motivations of AI for DR screening [16].

#### 3.4.5 Translation Across Contexts

The clinical context was described in numerous studies. Vaghefi et al. [27] deployed their AI technology in urban tertiary and rural primary care settings with favourable outcomes. Wijesinghe et al. [29] described that AI technologies for DR screening can be conducted in mobile healthcare centres. Scheetz et al. [25] described the deployment of an offline AI system across endocrinology outpatient clinics and Aboriginal medical services with clinical accuracy and stakeholder acceptance.

#### 3.4.6 Cost

Cost to the administrating practice was discussed as a barrier in two studies, citing start‐up cost concerns with AI implementation [16, 28]. On the other hand, cost efficiency was reported as advantageous in two studies [16, 26]. Held et al. [16] report low acquisition costs, the potential for practices to borrow screening devices, the simplicity of billing procedures and financial incentives in the context of Germany's health system as potential enablers.

### 3.5 Healthcare Professional

#### 3.5.1 Demand for the Profession

One study reported that 66% (*n* = 203/309) of questionnaire respondents, a cohort of ophthalmologists and physicians, believed AI would decrease the required workforce [14]. In this study, 47% (*n* = 147/309) of respondents agreed with a statement that AI would be a competitor to clinical practitioners, implying potential negative effects on the labour market [14].

#### 3.5.2 Reputability

One study described threats perceived by clinician respondents to inter‐professional relationships due to fewer referrals resulting from AI‐enabled screening occurring at primary care, while others recognised a potential to fortify inter‐professional relationships through the cooperation of services [16]. Concerning enablers, one study described that a general practice (GP) may beneficially experience a gain to their reputation resulting from the adoption of novel AI technologies, through projecting a modern image [16]. Apprehensions relating to the question of medical liability and information security were reported across three studies [16, 25, 28]. Professional competence was discussed in one study, reporting a perception that reliance on AI poses a danger to patients and places the competencies of doctors at risk [16].

#### 3.5.3 Clinical Experience and Expertise

Experience, or lack thereof, both in the clinical field of eye health and with other novel health technologies was reported to influence the adoptability of AI for DR screening. Clinical expertise and experience were described favourably in three studies [14, 16, 17]. In one paper, increasing years of clinical experience were observed to increase subjective ratings of ‘above average’ or ‘excellent’ knowledge of AI [14]. Experience with digital health, for instance e‐health, was reported in two studies, observing that prior experience fostered positive attitudes towards AI adoption and increased likelihood to perceive digital health interventions as assets to clinical efficiency, while inexperience was a barrier [14, 16]. Meanwhile, in one study, no notable differences were identified between primary care and eye‐care sites in AI system performance for the metrics of sensitivity, specificity, and imageability for mild to moderate DR and vision‐threatening DR [17]. At the primary care sites, where most operators had no prior imaging experience, the imageability rate was 90% on the first attempt and 97% under a dilate‐if‐needed protocol, compared to 98% at eye‐care sites [17]. One study outlined operator factors as contributing to ungradable fundus photos, while in a different study, all images were deemed gradable regardless of the device operator [24, 27].

#### 3.5.4 Education

Investment in education and support for staff was reported in several studies. Relating to openness towards novel practices, Held et al. [16] reported that learning and applying new skills, as well as the requirement to change established practice processes, act as barriers to implementation. Barakat reported that only 48% (*n* = 148/309) of respondents agreed that their organisation ‘*is likely to invest in AI in the clinical practice for DR in the next five years*’, and that 54% (*n* = 168/309) agreed the organisation is likely to provide AI training to healthcare workers in the next 5 years [14]. The training of staff without prior retinal imaging experience such as technicians, nurses and other non‐medically trained clinicians to operate the technology, capture images and trouble‐shoot was described in five studies [15, 17, 20, 22, 25]. Held et al. [16] described that AI would beneficially enable medical assistants to capture retinal images, affording doctors more time for other clinical responsibilities. The time to train staff to operate the technology ranged from 1 h to 2 weeks [16, 18, 19, 30]. However, the lack of available opportunities to utilise the experimentally adopted system was a barrier to uptake [25]. Lack of available support to answer questions or solve medical or technical problems was cited as a potential barrier in one study, suggesting that the accompaniment of a device representative during the initial operation would be facilitative [16]. Insecurity or lack of knowledge regarding ophthalmological topics and discussion of abnormal results to patients were raised as potential barriers in the two studies [16, 25].

#### 3.5.5 Attitudes and Beliefs

Two studies discussed the subjective attitudes of health professionals concerning AI for DR screening. Positive subjective assessments of AI technologies were reported, with 94% (*n* = 194/207) of respondents reporting being either satisfied or extremely satisfied in one study [25]. In the same study, 93% (*n* = 193/207) of participants were likely or extremely likely to utilise the system again [25]. Meanwhile, scepticism regarding the quality of the assessment was reported in another study [16].

### 3.6 Healthcare User

#### 3.6.1 Impact on Patient Outcomes

Holistic clinical assessment was identified as a potential barrier to adoption; the inability of the implemented AI system to detect non‐DR pathologies was noted in four studies [21, 24, 26, 27]. Conversely, beyond DR, diabetic maculopathy was detectable by AI in three studies, which prompted referral or further investigation at point‐of‐care [24, 26, 27]. Positive impacts on patient welfare by way of improved care delivery were reported in four studies [14, 16, 21, 28]. Barakat found that 85% (*n* = 263/309) of respondents agreed AI would increase the detection of early‐stage DR and decrease the progression of advanced DR [14]. One study noted that AI would facilitate more targeted referrals to specialists [16]. One study reported that the primary care context of the GP may increase the number of patients screened for DR and therefore rates of detection [16]. Reducing loss to follow‐up was discussed by Liu et al. [21], wherein 55% (*n* = 51/92) of patients referred by the AI system followed up with appointments at 12 months, compared to 19% (*n* = 182/974) of the historical cohort. Two studies described that the implementation of AI was an opportunity to provide education, aiming to empower patients with knowledge and to improve motivation to manage their condition [21, 28].

#### 3.6.2 Patient Perception and Experience

Patient preference with the implementation of AI was explored in several studies. Acceptability of the technology was discussed as a factor in accepting the diagnosis and improving adherence to DR screening [17, 19, 28]. Satisfaction was surveyed in one study which reported that 67% (*n* = 50/75) of patients were ‘very satisfied’ with the DR screening performed by AI [30]. In one study, 22% (*n* = 12/55) of patients preferred manual screening, of whom six cited trust as the reason for this preference [19]. Patients reported immediacy of screening results as favourable in two studies [19, 30]. The provider at which screening occurred was discussed in one study, wherein 68% (*n* = 51/75) of respondents expressed a preference to have screening performed in primary care [30]. Three studies noted healthcare access as an enabler, citing that primary care screening was favourable, possibly conferring cost benefits and that AI would improve accessibility to screening and follow‐up at the patient's convenience [14, 16, 17]. Meanwhile, cost to the patient was raised as a potential obstacle to implementation, with 32% of patients (*n* = 24/75) willing to pay an average of €28 for an AI screening test in one study [30].

### 3.7 Information Technology

#### 3.7.1 Diagnostic Performance

Objective assessments of deployed AI systems relating to imageability and efficacy were reported in numerous studies. Images deemed ungradable ranged from 3% to 24%, resulting in an inability to form a diagnostic conclusion or the need to refer [19, 23, 31]. A barrier in one study was the discrepancy observed for images deemed ungradable by the AI algorithm compared to the ophthalmologist (36% vs. 16%) [30]. A discrepancy was reported between AI and manual diagnosis for referrable images (41% vs. 24%), resulting in a relative increase of referrals by 72% when using the AI [30]. Automated assessment of image quality was regarded favourably by one study, and the lack thereof a barrier in another [16, 30]. Regarding patient factors, cataract or other media opacities were reported as impeding image quality and diagnosability in six studies [16, 21, 22, 23, 24, 31]. Small pupil size was a barrier to the capture of gradable images in two studies [21, 31]. Similarly, two studies describe that non‐mydriatic photos affected gradability [16, 17]. Diverse population characteristics posed a challenge to implementation, as described in one Australian study implementing AI that was trained on retinal images derived from a Chinese population; lower specificity was observed for Aboriginal Australian patients compared to non‐Aboriginal Australian patients [25]. Two studies described patient age as adversely affecting image quality and therefore impeding AI assessment, with Mehra et al. [22, 30] observing that 94% of images were deemed gradable in patients under 70 years compared to 85% for patients aged over 70 years. However, one study described the technology's lack of patient bias for gender, age and ethnicity, and operator bias for clinic, clinician and camera as facilitating adoption [27]. A second study discussed generalisability to different ethnic groups as a facilitator [15]. Concerning retinal image protocols, the region of the retina imaged was observed to modify image quality, with the region temporal to the macular adversely impacting AI assessment [30]. Conversely, one study reported that the site of the retina imaged did not negatively affect image gradability [27]. Regarding the diagnostic capacity of AI, one study reported that multiple eye disease diagnosis capabilities would act to enable implementation [16]. The incorrect diagnosis of exudate for large pachydrusen and small and hard drusen was cited as a barrier due to inconsistent DR grading by the system used in one study [27]. One AI device performed with low specificity, producing many false positives and potentially causing unwarranted anxiety for patients [26]. Artefacts such as a central halo generated by one camera and an artefactual dot in another camera were tolerated by the AI system used in one study [27]. One article reported concerns regarding the way by which AI generates diagnostic conclusions [16]. Transparency in the AI algorithm and arrival at a diagnostic conclusion were reported as favourable by Vaghefi et al. [26], whose tested algorithm creates attention maps. Doubt concerning AI development and the requirement for ongoing technological updates and upgrades was reported in one study [28].

#### 3.7.2 Information Technology Infrastructure

Several studies discussed information systems. Simple software installation and integration into pre‐existing practice information systems were reported to facilitate implementation [16]. In one study, an ensemble AI model, as opposed to a singular model, required increased computational power, telecommunication support and running time [15]. In another study, the use of a cloud‐based system enabling direct transmission of data to an ophthalmologist was viewed favourably [16]. While in one study, low‐speed internet connectivity delayed image upload and receipt of results, in another study, an offline AI system not depending on internet connection was favourable [24, 25]. Equipment shortcomings such as dirty camera lenses obscuring the retinal image were reported in two studies [18, 30]. Camera‐related factors were cited as reasons for AI systems being non‐diagnostic in one study, which identified different image gradeability across different camera models used [24]. Contrastingly, the model of camera used was inconsequential for image gradability and AI diagnostic capability in four studies [24, 25, 27, 31].

#### 3.7.3 Systems Interface and Usability

Systems interface and usability were identified as factors for successful implementation in one study, reporting a user‐friendly screening device and software as facilitative factors [16]. Meanwhile, two studies reported AI system functions such as input and comprehension errors, as well as overwriting of images for new image attempts as barriers [29, 30]. Enabling implementation, the technology utilised in one study could self‐edit photos to improve functionality and resize images to standardise dimensions [29]. One study reported that a semi‐automated device that identifies the patient's eye, centres the camera on the pupil, and follows a pre‐specified imaging protocol enabled deployment [30]. One study favourably reported that 92% of all images captured were in photographic focus [30].

## 4 Discussion

While AI systems have been deployed in various clinical settings with widely reported accuracy for DR diagnosis, this review synthesises existing literature describing the barriers and enablers to its uptake in clinical practice as explored by 18 studies. The World Health Organisation's *World Report on Vision* suggests a guiding framework for the development of integrated people‐centred eye‐care, comprising four strategies: engaging and empowering communities, reorienting care models upon strong primary care, coordinating services, and creating environments for integrating eye‐care in national plans and health systems where the workforce meets population needs [32]. The findings from this scoping review indicate factors within the healthcare system, the healthcare professional, the healthcare user and information technology can either enable or act as a barrier to the uptake of AI in DR screening; it is upon these ideas that strategies can be created to strive for better eye‐care.

All of the selected studies reported on healthcare system factors such as efficiency, protocolisation, referrals and costs associated with implementing AI for the screening of DR [14, 15, 16, 17, 18, 19, 20, 21, 22, 23, 24, 25, 26, 27, 28, 29, 30, 31]. Concerning a technological advancement designed to enhance health service delivery, that this domain received universal attention from the included studies is unsurprising. Regarding providers, our findings identify that attitudes and beliefs within the industry, effects on demands for the profession, the education of providers and professional reputability are among factors that the literature critiques in its examination of the clinical applicability of AI for DR screening [14, 16, 17, 18, 19, 20, 22, 24, 25, 27, 28, 30]. As members of society entrusted with the health of service consumers, the education of providers was most frequently assessed in this domain—while an enabler in some studies discussing the sharing of responsibilities among health practitioners, education was a barrier in others citing insufficient or unsupported training [15, 16, 17, 20, 22, 25]. Importantly, several studies report on patient‐related factors impacting the applicability of AI in DR screening, with improved longitudinal care and more targeted referrals among the enablers noted in seven studies compared to the four which identified barriers within the outcomes achieved by the AI intervention [14, 16, 17, 19, 21, 24, 26, 27, 28, 30]. Finally, information technology considerations were discussed in terms of factors influencing clinical applicability, most frequently concerning diagnostic performance, then infrastructure, then usability [16, 17, 18, 19, 21, 22, 23, 24, 25, 26, 27, 28, 29, 30, 31].

As a novel health intervention, AI for DR screening is well‐positioned to be considered using an implementation science lens. This is particularly important, as barriers and enablers can be two sides of the same coin, depending on the context, stakeholder perspective and how a particular factor is managed. For example, education is a barrier when unfulfilled, and an enabler when harnessed through training opportunities [15, 16, 17, 20, 22, 25]. Similarly, information technology can act as an enabler when it is user‐friendly and accurate but becomes a barrier when it is expensive or difficult to use [16, 30]. It is in this context that implementation science examines the factors influencing the uptake of new health technologies, therein forming frameworks to predict and guide conditions required for successful implementation, which for AI is demonstrably ineffective when approached passively [33]. Rather, implementing such technologies should be purposive and evidence‐based [34]. An abundance of literature has been published about computer science sustaining AI in health applications; however, implementation science studies examining its deployment in real‐world healthcare settings is lacking [34]. Better understanding these gaps, and work that aims to address them, can assist in successfully and sustainably embedding such technological innovations in healthcare.

Notably, factors influencing uptake of digital health innovations are not exclusive to AI. Reports of e‐health, that is using information technology to support a health service, for diverse applications in ophthalmology are increasing [35]. Korot et al. [36] investigated the use of smartphone applications for home‐based vision monitoring, reporting on factors influencing compliance and uptake including patient satisfaction, usability, age and level of comfort with modern technologies. As identified in our study, the healthcare user, in particular the patient perception and experience, are both enabling and inhibiting for DR screening with AI. Another application of e‐health is telemedicine, enabling clinical interactions to occur between geographically distant healthcare providers and users [35]. The literature reports costs, technological infrastructure, personnel training, information confidentiality and security, and medicolegal issues as barriers to its implementation in ophthalmology; with one study suggesting national policies may strategically support uptake and combat financial barriers [37, 38]. Our review similarly identified such healthcare system, provider and information technology factors as influencing the applicability of AI for DR screening.

Beyond ophthalmology, AI has been deployed for radiological and dermatological applications [39, 40]. Reinforcing our findings that the attitudes and beliefs of healthcare professionals are influential, a scoping review by Eltawil et al. [39] investigating the barriers and enablers for AI use in radiology describes lack of trust, impacts on demand for the profession and scarce evidence describing the effect of AI on the workflow as barriers; educating health professionals and establishing regulatory frameworks and guidelines are suggested strategies to challenge them.

As our review identifies, the healthcare system and professionals alike are key factors influencing the uptake of AI for DR screening, which has implications for future workplace planning. For instance, pressures including training bottlenecks for junior physicians, time constraints and patient loads contribute to the demands placed on specialist eye surgeons [41, 42, 43]. Improvements in coordinating health resources, demonstrably facilitated by AI use in DR screening, may promote sustainable, far‐reaching delivery of eye‐care.

This study identifies that AI is deployed across various healthcare settings for the screening of DR, imperatively offering the ability to improve screening scope. In settings experiencing resource constraints, such as remote communities in Australia, improved access to DR screening was observed in piloted outreach services [44]. Indeed, for rurally‐ and remotely‐delivered DR screening, recruiting professionals such as outreach coordinators, optometrists, orthoptists and ophthalmologists yields great potential [9]. Accordingly, the utility of AI for DR screening may offer opportunities to address disparate health outcomes in low‐resource settings or disproportionately‐affected patients, such as for Aboriginal and Torres Strait Islander populations in Australia where eye screening service delivery decreases with increasing remoteness [45]. Furthermore, the study by Bellemo et al. [15] affirmed the effectiveness of AI deployed in a DR screening program in Zambia, a low‐resource country, and with operation by non‐medical staff. Training adjunctive clinical staff to administer AI‐based DR screening alleviates pressures on the medical profession, profoundly impacting screening processes [15, 16, 17, 20, 22, 25].

Understanding clinical applications of AI in other specialties, in addition to understanding how novel health applications have been successfully implemented, will assist stakeholders to adopt strategies favouring the use of AI in DR screening.

With a scarcity of literature reporting the barriers and enablers influencing clinical implementation of AI for DR screening, further research is warranted to harness this revolutionary health innovation.

### 4.1 Strengths, Limitations and Recommendations

This scoping review was informed by transparent and evidence‐based protocols as well as an experienced research team in review methodology. An academic librarian guided the search strategy. The inclusion criteria broadened the scope of papers retrieved, capturing various study methods to maximise the barriers and enablers identified. This review also has limitations. Restricting the studies to English creates potential for language bias. However, the included studies originated from a diverse range of countries, many where English is not the primary language. Therefore, the findings from this review are likely to be generalisable on a global scale. Given the heterogeneity of the included studies, barriers and enablers were categorised broadly into four domains and not analysed by characteristics such as the clinical setting and AI intervention used. With a developing evidence base in this space, future research could explore how different clinical settings and AI interventions influence these factors.

In identifying the barriers and enablers influencing the clinical applicability of AI for DR screening, this study highlights recommendations from workforce, clinical and industrial perspectives that can be fostered for its real‐life deployment.

### 4.2 Conclusion

Our review findings provide understandings on the barriers and enablers of artificial intelligence for diabetic retinopathy screening. Translating the knowledge of these systems, provider, consumer and technological factors informs clinical strategies, from optimising the clinical environment through to educating clinical providers. Understanding their action in enabling or challenging implementation, we call for deliberate and coordinated strategies to sustainably and effectively engage with this fifth industrial revolution that is AI for DR screening practices, which is of immense potential for disrupting eye health inequities.

## Conflicts of Interest

The authors declare no conflicts of interest.

## Data Availability

Data sharing is not applicable to this article as no new data were created or analysed in this study.

## Appendix A Exemplar Search Applied to OVID MEDLINE

1 (“artificial intelligence” or “deep learning” or “machine learning” or “supervised machine learning” or “unsupervised machine learning” or “augmented intelligence”).mp.

2 exp. Artificial Intelligence/

3 ((diabet* or proliferative or non?proliferative) adj4 retinopath*).mp.

4 (diabet* adj3 (eye* or vision or visual* or sight*)).mp.

5 Diabetic Retinopathy/

6 ((enable* or barrier* or facilitat* or limit* or promot* or problem* or challeng* or feasib*) adj4 (applica* or translat* or implement* or utili* or deploy* or clinic* or practic* or deliver* or adopt*)).mp. [mp = title, abstract, original title, name of substance word, subject heading word, floating sub‐heading word, keyword heading word, organism supplementary concept word, protocol supplementary concept word, rare disease supplementary concept word, unique identifier, synonyms] 385 590

7 3 or 4 or 5

8 1 or 2

9 6 and 7 and 8
